# Bisphenol A Promotes the Progression of Colon Cancer Through Dual-Targeting of NADPH Oxidase and Mitochondrial Electron-Transport Chain to Produce ROS and Activating HIF-1α/VEGF/PI3K/AKT Axis

**DOI:** 10.3389/fendo.2022.933051

**Published:** 2022-07-04

**Authors:** Tianyi Xia, Junnan Guo, Bomiao Zhang, Chengxin Song, Qunye Zhao, Binbin Cui, Yanlong Liu

**Affiliations:** Department of Colorectal Surgery, Harbin Medical University Cancer Hospital, Harbin Medical University, Harbin, China

**Keywords:** bisphenol A, colon cancer, progression, ROS, HIF-1α/VEGF/PI3K/AKT axis

## Abstract

Bisphenol A (BPA) is a high-production-volume industrial chemical. Despite recent research conducted on its carcinogenicity, its role in the development of colon cancer (CC) has been rarely studied. This study aims to evaluate the effects of BPA on the migration and invasion of CC cells. First, we clinically verified that patients with CC exhibit higher serum BPA level than healthy donors. Subsequently, different CC cell lines were exposed to a series of BPA concentrations, and the migration and invasion of cells were detected by the wound healing test and transwell assay. Finally, N-acetyl-L-cysteine (NAC) and siHIF-1α intervention was used to explore the effects of ROS and HIF-1α on cell migration and invasion, respectively. The results demonstrated that the occurrence of BPA-induced migration and invasion were dependent on the dose and time and was most pronounced in DLD1 cells. ROS production was jointly driven by NADPH oxidase (NOX) and mitochondrial electron-transport chain (ETC). Furthermore, the intervention of NAC and siHIF-1α blocked the HIF-1α/VEGF/PI3K/AKT axis and inhibited cell migration and invasion. In conclusion, our results suggest that BPA exposure promotes the excessive production of ROS induced by NOX and ETC, which in turn activates the HIF-1α/VEGF/PI3K/AKT axis to promote the migration and invasion of CC cells. This study provides new insights into the carcinogenic effects of BPA on CC and warns people to pay attention to environmental pollution and the harm caused to human health by low-dose BPA.

## Introduction

Bisphenol A (BPA) is a raw material of polycarbonate plastic and epoxy resin, which is largely used to produce consumer products, including food containers, toys, medical materials, water pipes, and thermal paper (1). BPA can be released from various related consumer products and deposited in the environment. A review of more than 500 peer-reviewed papers showed that BPA is widely distributed in soil, air, surface water, and sewage sludge (2). BPA can cause toxicity in tissues and organs even at very low dose levels. An increasing number of studies of the underlying mechanisms of BPA exposure over the past decade have indicated that a wide variety of BPA doses increase reactive oxygen species (ROS) generation, break the antioxidant balance, and promote changes in many cell signaling pathways related to oxidative stress, including apoptosis, autophagy, and inflammation (3). BPA is related to increased cancer risk, which is manifested by its effects on tumor cell proliferation, invasion, angiogenesis, and epigenetic pathway (4). Shi et al. used NCM460 cells to demonstrate that BPA exposure promotes the migration of human normal colonic epithelial cells (5). BPA also modulates the colorectal cancer (CRC) protein profile and promotes the metastasis of SW480, LoVo, and HCT-116 cells by inducing epithelial-to-mesenchymal transitions (EMTs) (6). A recent study showed that BPA not only increases the incidence and poor outcomes of common cancers but also induces many tumor cells to develop drug resistance to classical chemotherapy drugs (7).

Metastasis is a characteristic hallmark of cancer, resulting in 90% of cancer-related deaths. Accumulative evidence indicates that ROS is a major participant in the development of multiple cancers in terms of regulating the metastatic properties of cancer cells (8). The special metabolic state of tumor leads to its long-term hypoxia. Under the hypoxia condition, ROS produced by the mitochondrial electron-transport chain (ETC) is significantly increased (9), followed by the stimulation of high-expression and transcriptional activity of HIF-1α (10). HIF-1α activation is the central link in regulating hypoxia response gene expression. Numerous studies have shown that HIF-1α overexpression is highly related to tumor migration and invasion (11). To the best of our knowledge, various cytokines are produced by cancer cells, including VEGF. VEGF expression is majorly adjusted at the transcriptional level by HIF-1α in hypoxia state (12). On one hand, VEGF secreted by tumor cells and an extracellular matrix can promote angiogenesis and tumor progression (13). On the other hand, VEGF can promote tumor evolution through non-angiogenic mechanisms (14). PI3K/AKT is the main downstream cellular pathway mediating the biological effects of VEGF (15), and the VEGF/PI3K/AKT signaling pathway plays a significant role in various cellular processes, including migration and invasion (15). For example, preventing the activation of VEGF/PI3K/AKT pathway inhibits the migration and invasion of hepatocellular carcinoma cells (16).

CRC is the third most common type of cancer globally, and colon cancer (CC) accounts for approximately 70% of all forms of CRC. In addition, dietary and environmental factors are important causes of death in more than 80% of CC cases (17). Considering the high detection rate of BPA in ecological and human samples and the high fatality rate of contemporary human CC, the study of the toxicological mechanism of BPA in the occurrence and development of CC is significant. However, the effects of BPA exposure at different concentrations on the migration and invasion of CC cells, as well as the roles of ROS and HIF-1α in this process, have not been completely clarified. In this study, we clinically verified that patients with CC exhibit a higher BPA level in blood than healthy people. In addition, we treated various CC cells with different BPA concentrations to select the appropriate cell line as the research object. A wound healing test and a transwell assay were conducted to evaluate cell migration and invasion. Meanwhile, we intervened DLD1 cells exposed to BPA with N-acetyl-L-cysteine and siHIF-1α to further illustrate the relationship between ROS/HIF-1α and the migration and invasion of CC cells under BPA exposure. Our study results not only provide a theoretical basis for developing new prognostic molecules and appropriate methods for treating BPA-related cancers in the future but also deepen the understanding of the tumor-promoting signal pathway.

## Materials and Methods

### Clinical Samples

All studies were performed with the approval of the Ethical Committees of Harbin Medical University Cancer Hospital. Informed written consent was obtained from all participants. We recruited healthy individuals (n = 60) and treatment-naive patients (n = 60) who were diagnosed with CC based on endoscopic and histopathological criteria. Their blood samples were collected and centrifuged at 3500 rpm for 15 min to obtain the serum, and then, the BPA concentration in the serum was detected using the ELISA kit (Jingmei Biotechnology Co., Ltd., Jiangsu, China).

### Cell Culture and Treatment

The SW620, HT-29, HCT116, and DLD1 cells were stored in a complete medium containing 10% fetal bovine serum (FBS, Biological Industries, Kibbutz Beit Haemek, Israel) and 90% RPMI 1640 (Thermo Fisher Scientific, Waltham, MA), and cultivated in a humidified incubator with 5% CO_2_ at 37°C. BPA (B802575, Macklin Biochemical Co., Ltd., Shanghai, China) was dissolved in dimethyl sulfoxide (DMSO, Minibio, China) to produce a 1M stock solution. Then, the cells were divided into a control group and different treatment groups: the control group was only cultured in a complete medium, and the other groups were treated at different BPA concentrations (1 × 10^-7^, 1 × 10^-6,^ 1 × 10^-5^, 1 × 10^-4^, and 1 × 10^-3^ mM) and different time points (0 and 24 h). NAC (Beyotime, Shanghai, China) was dissolved in DMSO and diluted with medium to produce a working concentration of 2 mM, and pre-incubated with DLD1 cells for 10 h to inhibit ROS production. Additionally, the NAC-alone treatment group was used to eliminate the inhibitory effect caused by self-toxicity. Three small interfering RNAs (siRNAs) targeting HIF‐1α (siHIF‐1α) and negative-control siRNA with a scrambled sequence (siNC) were synthesized by RiboBio Co., Ltd. (RiboBio, China). The target sequence was as follows: siHIF-1α-1: GGAACATGATGGTTCACTT, siHIF-1α-2: CTACCCACATACATAAAGA, siHIF-1α-3: CCAGCAACTTGAGGAAGTA. DLD1 cells were transfected with 3 μL of 20 μM siRNAs and 3 μL of Lipofectamine 2000 reagent (Invitrogen) in 1 mL of Opti-MEM for 24 h. The supernatants were removed and replaced with a fresh culture medium, followed by 24 h of culture with or without BPA.

### Wound Healing Assay

Six parallel lines were drawn on the back of a 6-well cell-culture plate, in which DLD1 cells were inoculated at a density of 5 × 10^5^ cells/well to form cell monolayers. The cells were “scraped” in a direction perpendicular to the parallel line with the tip of a 200 μL sterile pipette and rinsed with PBS to remove cellular debris. Subsequently, the wounded cell monolayers were exposed to different BPA concentrations (1 × 10^-7^, 1 × 10^-6,^ 1 × 10^-5^, 1 × 10^-4^, and 1 × 10^-3^ mM) and cultured in an incubator. The scratch was imaged by an optical microscope at 0, 12, and 24 h, and the distance between the edges of six scratches intersecting with the parallel lines was measured. The wound closure rate (of control) = distance (treated sample)/distance (control) − 1.

### Transwell Invasion Assay

Transwell chambers containing matrigel were placed in a 24-well cell-culture plate. After rehydration of the matrigel with a serum-free culture solution, DLD1 cells stimulated by different BPA concentrations were prepared into a single-cell suspension with 5×10^4^ cells by using a culture medium containing 2% FBS, which was added to the upper chamber. Then, a 500 μL complete culture medium containing 10% FBS was added to the lower chamber. After being cultured in a cell incubator containing 5% CO_2_ at 37°C for 24 h, the cells on the upper surface of the gel and polycarbonate membrane were carefully wiped off with cotton balls. Then, inserts were washed two times with PBS and fixed in 4% paraformaldehyde for 20 min. Following staining with 0.1% crystal violet for 15 min, the inserts were observed and photographed under an inverted microscope at a magnification of 20×. Subsequently, the chambers were placed in a 24-well plate containing 500 μL 33% acetic acid and shaken for 10 min to dissolve the crystal violet completely. The OD value was then detected at 570 nm by using a microplate reader, which indirectly reflected the number of migrating cells. Cell migration rate (of control) = OD (treated sample)/OD (control) − 1.

### Evaluation of Oxidative Stress

DLD1 cells were inoculated in a 6-well cell-culture plate at a density of 5 × 10^4^ cells/well and allowed to incubate at 37°C for 24 h under the specified treatment. Then, the cells were washed and incubated in RPMI 1640 with a 10 μM DCFH-DA probe (Nanjing Jiancheng Bioengineering Institute, Nanjing) at 37°C for 20 min. After washing three times with PBS to remove the probe residue, the fluorescence images of DLD1 cells were obtained using a fluorescence microscope (Olympus Corporation, IX53), before which the whole operation was performed in dark. Finally, the fluorescence intensity was quantified using the ImageJ software. The contents of MDA and LPO and the activities of T-SOD and GSH-PX were detected according to the manufacturer’s instructions provided with the oxidative stress kit (Nanjing Jiancheng Bioengineering Institute, Nanjing).

### qRT-PCR Assay

The total RNA (1 μg) extracted from DLD1 cells was reverse-transcribed into cDNA using a cDNA first-strand synthesis kit (Bioer Technology Co., Ltd., Hangzhou, China) as per the manufacturer’s protocol. The details of the primers used in this test are listed in **Supplement-Table 1**. Continuous fluorescence measurements were performed on Quant Studio 3 (Thermo Fisher, Lithuania). The mRNA expressions were quantified using the comparative 2^-ΔΔCt^ method. The β-actin were used as endogenous control.

### Western Blotting Analysis

DLD1 cells were seeded in 6-well cell-culture plates at a density of 1 × 10^6^ cells/mL following NAC pretreatment or/and BPA exposure. The cells were then rinsed two times with PBS and lysed in a cold RIPA lysis buffer containing 1 mM PMSF. The supernatant was collected through centrifugation at 4°C and 12000 rpm for 15 min, and the total protein concentration was determined using the BCA method. In addition, SDS-polyacrylamide gel electrophoresis was performed, and the separated proteins were transferred to the nitrocellulose membrane, which was blocked in 5% skim milk powder at 37°C for 2 h. Next, the membrane was washed with TBST three times and incubated with the primary antibody at 4°C overnight, followed by the incubation of the HRP-bound goat anti-rabbit IgG secondary antibody at room temperature for 2 h. The antibodies used in this test are shown in **Supplement-Table 2**. Finally, the signal intensity was detected using Image J software (National Institutes of Health). β-actin was used to verify the equal protein loading.

### Molecular Docking

Molecular docking was applied to predict the likely binding modes of a ligand (BPA) with NOX and ETC of 3D structure. Briefly, 3D structure for the NOX and ETC targets were chosen from PDB Bank (http://www.rcsb.org/pdb/home/home.do), and then, the selected NOX, ETC and prepared BPA ligand were output for the docking setup. For ligand–protein docking, the AutoDock 4.2.6 software was used to remove the water molecules, discard any structures that lacked the biologically necessary cofactors, and indicate hydrogens. Then, PyMol 2.2.0 was used to identify the most favorable mode of binding BPA to NOX and ETC. After optimization, the optimal binding mode was output.

### Statistical Analysis

Statistical analyses and image generation were performed using GraphPad Prism 8.0 (GraphPad Software, Inc.). The results were recorded as means ± SD, where *p* < 0.05 showed significant difference from the corresponding control group. For two-group comparisons, unpaired two-tailed t-tests were performed. For multiple comparisons, one-way analysis of variance, followed by Tukey’s multiple-comparisons test method, was conducted to determine differences in the means.

## Results

### Exposure to BPA With an Increased Risk of CC

**Figure 1:**
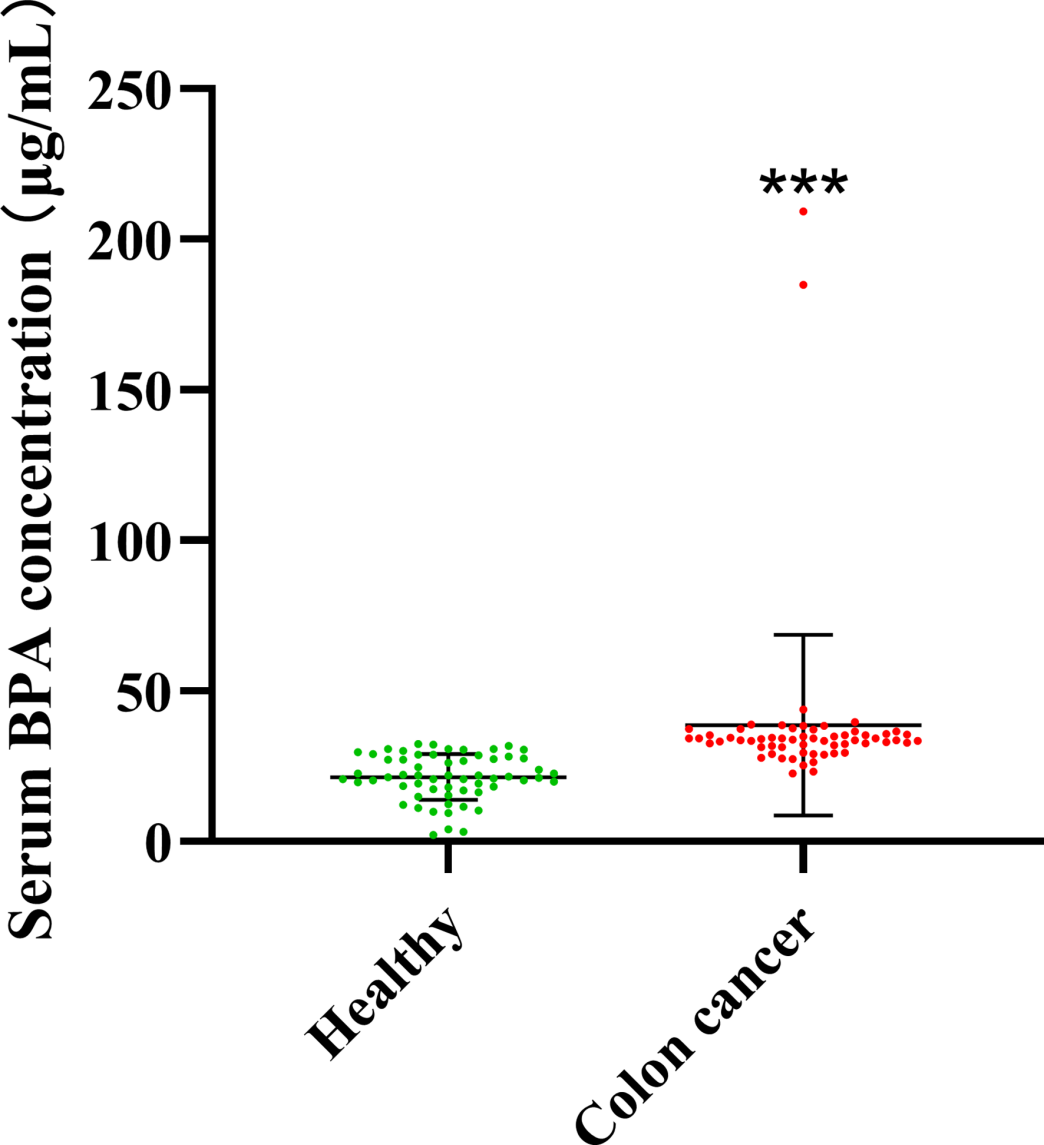
Concentration of BPA in serum from healthy donors and individuals with colon cancer. Values are expressed as means ± SD (n = 60), compared with the healthy group, ***p < 0.001.

To investigate whether CC onset and BPA accumulation in the body are related, we measured the BPA content in the serum of healthy donors (n = 60) and that in patients with CC (n = 60). To avoid the effects of therapeutic drugs and variations in disease severity, we obtained the blood samples from recently diagnosed, untreated individuals with CC. According to the results, higher concentrations of BPA were found in the serum of individuals with CC than in healthy donors (**Figure 1**).

### BPA Augments the Migration Ability of Different CC Cells

**Figure 2:**
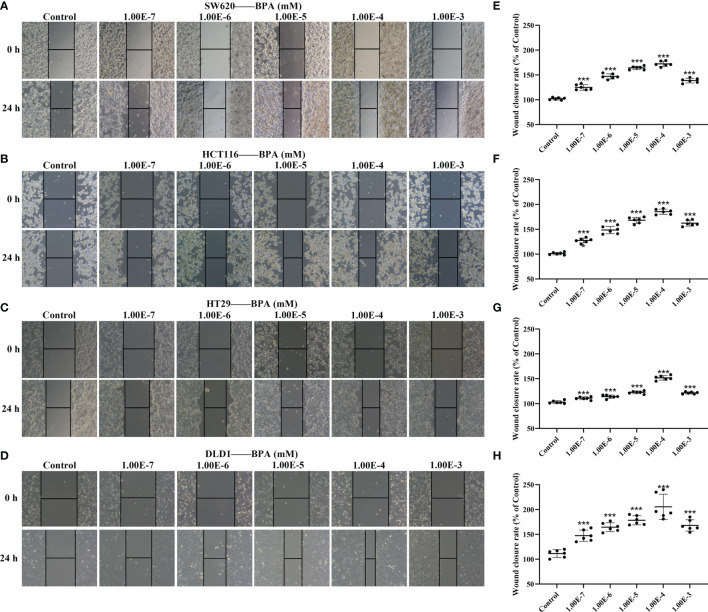
The comparison of BPA exposure on the migration ability of different colon cancer cells. Representative images of cell migration of SW620 **(A)**, HCT116 **(B)**, HT29 **(C)** and DLD1 **(D)** cells under different concentrations of BPA. Quantitative analysis of wound closure rate corresponding to cell migration of SW620 **(E)**, HCT116 **(F)**, HT29 **(G)** and DLD1 **(H)** cells exposed to BPA, n = 6. Values are expressed as means ± SD, compared with the control group, ***p < 0.001.

To test whether BPA affected the migration ability of CC cells, we used wound healing assay to evaluate cell migration. According to the BPA content (0.1 nM to 10 µM) used in a previous research (18), the CC cells, including SW620, HCT116, HT29, and DLD1, were treated with BPA concentrations ranging from 10^-7^ to 10^-3^ mM for 24 h. While the representative results obtained from **Figures 2A–D** showed that the CC cells in the scraping area were gradually increasing, further quantitative analysis showed an inverted U-shaped relationship between BPA exposure and cell migration (**Figures 2E–H**). The minimum wound closure appeared at 10^–4^ mM exposure concentration, and the promoting effect of BPA on the migration of CC cells was found to be the most pronounced in DLD1 cells.

### BPA Increases Migration and Invasion in DLD1 Cells Through the HIF-1α/VEGF/PI3K/AKT Axis

**Figure 3:**
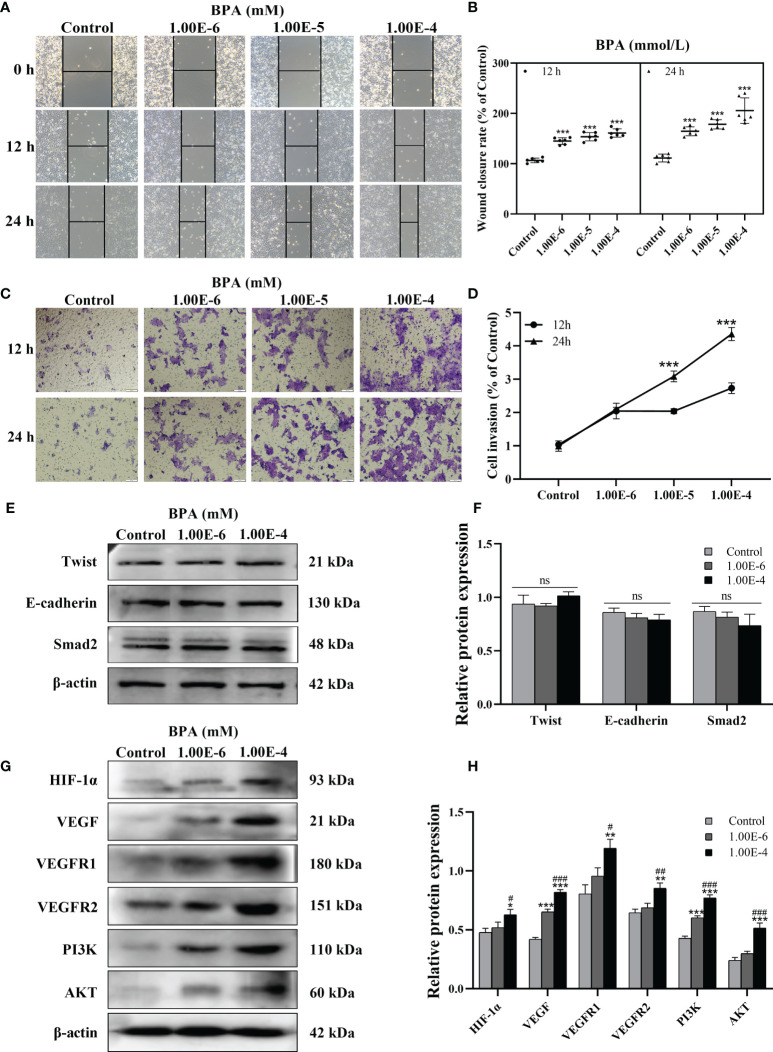
The effects of BPA on invasion, EMT, HIF-1α/VEGF and PI3K/AKT signaling pathway of DLD1 cells. Representative images of DLD1 cells exposed to BPA for 12 or 24 h in migration **(A)** and invasion **(C)** assay. Quantitative analysis of cell migration **(B)** and invasion **(D)** rate, n = 6. The protein expression of EMT **(E)** as well as HIF-1α/VEGF and PI3K/AKT signaling pathway **(G)** -related biomarkers in DLD1 cells. Quantitative analysis of expression of EMT **(F)**, HIF-1α/VEGF and PI3K/AKT signaling pathway **(H)** related protein by ImageJ Software, n = 3. Values are expressed as means ± SD, compared with the control group, *p < 0.05, **p < 0.01, ***p < 0.001. Compared with the 1.00E-6 BPA group, ^#^ *p* < 0.05, ^###^ *p* < 0.001. ns, no significance.

To further confirm the malignant development of CC cells under BPA-induced effects, DLD1 cells were treated with 10^-6^ to 10^-4^ mM BPA for 12 and 24 h, respectively. As shown in **Figures 3A–D**, the number of migrated and invaded cells exposed to BPA increased with the exposure concentration and peaked at the 10^-4^ mM level. In addition, the mobility and invasion of cells exposed to BPA at different concentrations for 24 h were higher than those of cells exposed to BPA at the corresponding concentration for 12 h. The results indicated that BPA enhanced cell migration and invasion in a time- and concentration-dependent manner. Because EMT, HIF-1α/VEGF, and PI3K/AKT signaling pathways tightly regulate tumor migration and invasion, we evaluated EMT progress by measuring the protein expression levels of Twist, E-Cadherin, and Smad2 in DLD1 cells. Interestingly, no significant changes in the aforementioned biomarkers were observed (**Figures 3E, F**). HIF-1α/VEGF and PI3K/AKT signaling pathways were assessed by detecting the translation levels of HIF-1α, VEGF, VEGFR1, VEGFR2, PI3K, and AKT. As illustrated in **Figures 3G, H**, all related proteins exhibited evident concentration-dependent increases upon BPA exposure. Overall, these data indicated that the BPA-induced cell migration and invasion had a positive association with the HIF-1α/VEGF/PI3K/AKT axis rather than EMT.

### Both NOX and ETC Are Involved in BPA-Promoted ROS Generation in DLD1 Cells

**Figure 4:**
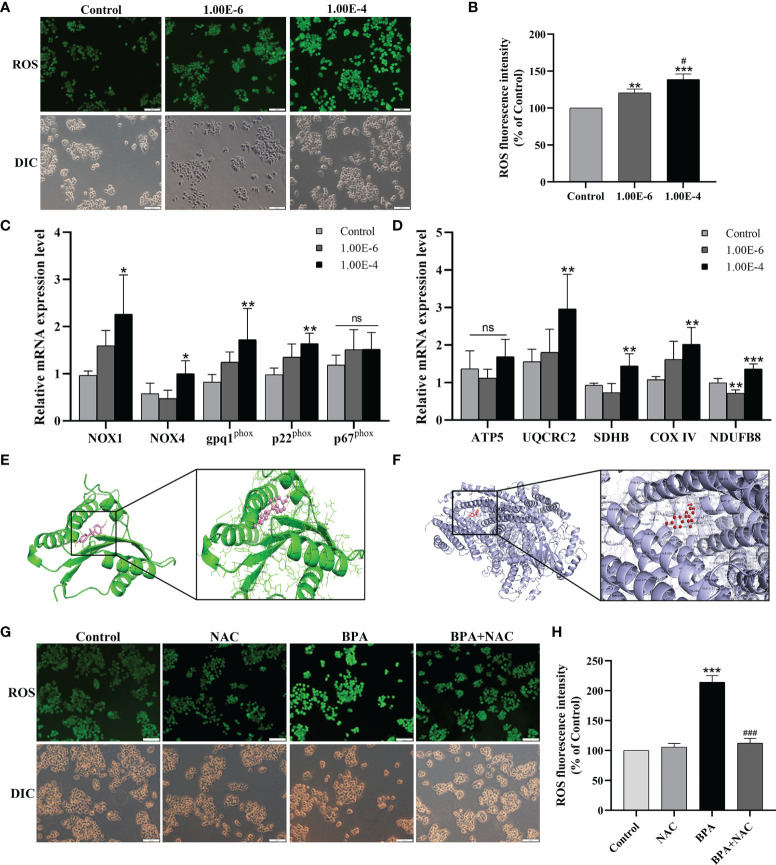
Both NOX and mitochondrial respiratory chain involved in the ROS generation promoted by the BPA in DLD1 cells. Oxidative stress makers of ROS **(A)** was evaluated in DLD1 cells, and quantitative analysis of ROS **(B)**, n = 6. The mRNA expression levels of NOX subunits **(C)** and mitochondrial respiratory chain complexs **(D)**, n = 6. **(E)** 3D docking model of BPA with NOX. BPA was shown as pink sticks and NOX as green ribbons. The hydrogen bonds are indicated by yellow dashed lines. **(F)** BPA with ETC protein in the region of binding site. ETC protein was represented in blue, and the structures of BPA were colored by red. The yellow dashed lines indicated hydrogen bond. **(G)** DLD1 cells were treated with 1 × 10^-4^ mM BPA for 24 h in the presence or absence of the ROS inhibitor NAC. The level of ROS **(G)** and quantitative analysis of ROS **(H)**, n = 6. Values are expressed as means ± SD, compared with the control group, *p < 0.05, **p < 0.01, ***p < 0.001. Compared with the 1.00E-6 BPA group, ^#^ *p* < 0.05, ^###^ *p* < 0.001. ns, no significance.

To investigate the oxidative stress of CC cells under BPA-induced effects, the oxidative stress indexes were analyzed. As shown in **Figures 4A, B**, the level of ROS in DLD1 cells increased significantly after exposure to BPA. Similarly, the contents of MDA and LPO in DLD1 cells exposed to 1.00E-4 mM BPA were also markedly higher than those in the Control group (**S-Figure 1**); however, the detection results of T-SOD and GSH-PX (**S-Figure 1**) showed the opposite trend to the above pro-oxidative index, and the enzyme activity both decreased obviously under the intervention of 1.00E-4 mM BPA, which indicated that BPA exposure promoted ROS production and excessive oxidative stress in DLD1 cells. Because intracellular ROS was mainly produced by ETC and NOX, to explore the source of BPA-induced ROS elevation in DLD1 cells, we first detected the mRNA level of NOX complex in BPA treatment groups at different concentrations (0, 10^-6^, and 10^-4^ mM). As shown in **Figure 4C**, we detected the transcription level of essential genes related to NOX. Except for p67^phox^, the mRNA level of other tested genes had evident concentration-dependent increases upon BPA-alone exposure. Then in **Figure 4D**, four of the five genes associated with the ETC complex (UQCRC2, SDHB, COX IV, and NDUFB8) also significantly increased after exposure to 10^-4^ mM BPA than those of the control group. In addition, we explained the possible interaction of BPA with NOX and ETC protein. The complete view of the 3D docking model of BPA with NOX was shown in **Figure 4E**. BPA mainly bounded to the groove on the surface of the NOX protein, where two molecules were closely integrated through chains within 3.0A. Additionally, **Figure 4F** showed the docking view of BPA in the binding site of ETC protein, where BPA mainly bounded to the groove inside ETC protein. In particular, BPA docked into ETC protein with its residues located in the inner region of the binding pocket. Therefore, based on the model assessment results, we affirmed high affinity between BPA and NOX, as well as the ETC protein. We next evaluated oxidative stress in DLD1 cells exposed to NAC or/and BPA. The results showed that NAC pretreatment can restore the ROS changes in ROS (**Figure 4G, H**), MDA, LPO, T-SOD, and GSH-PX (**S-Figure 1**) induced by BPA to the normal level. Moreover, NAC pretreatment significantly reversed the expression levels of the abovementioned genes in the BPA-exposed group (**S-Figure 2**). Overall, these results indicated the importance of ETC and NOX in regulating BPA-induced ROS production.

### ROS Scavenging Inhibits BPA-Induced Migration and Invasion of DLD1 Cells

**Figure 5:**
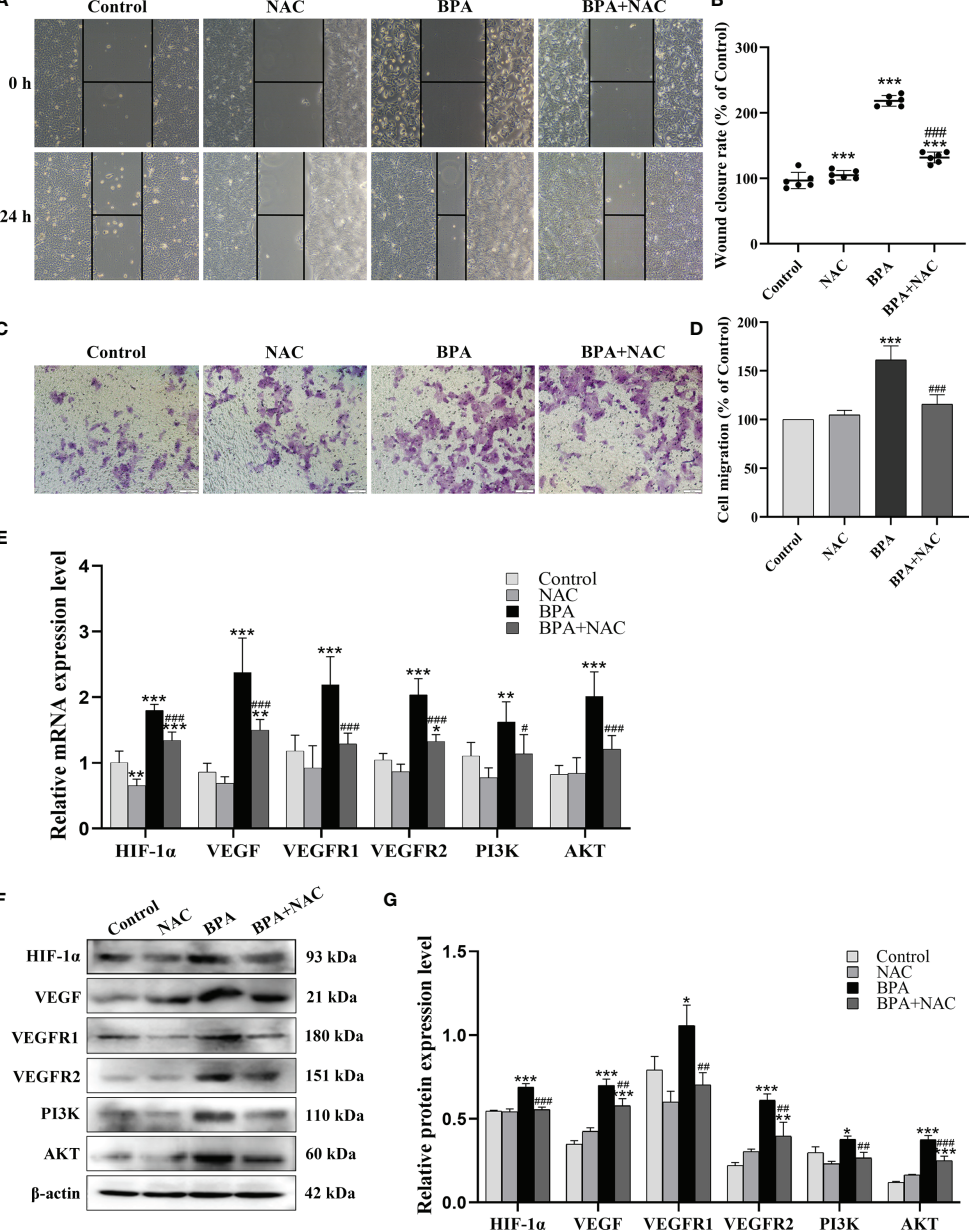
The role of ROS generation on the BPA-promoted migration, invasion, HIF-1α/VEGF and PI3K/AKT signaling pathway of DLD1 cells. Representative images showed the cell migration **(A)** and invasion **(C)** assay. Quantitative analysis of cell migration **(B)** and invasion **(D)** rate, n = 6. The mRNA **(E)** and protein **(F)** expression of HIF-1α/VEGF and PI3K/AKT signaling pathway-related biomarkers in DLD1 cells. **(G)** Quantitative analysis of protein expression associated with HIF-1α/VEGF and PI3K/AKT signaling pathway by ImageJ Software, n = 3. Values are expressed as means ± SD, compared with the control group, * *p* < 0.05, ** *p* < 0.01, *** *p* < 0.001; compared with the BPA group, ^#^ *p* < 0.05, ^##^ *p* < 0.01, ^###^ *p* < 0.001. ns: no significance.

Considering the critical role of ROS in cancer progression, we analyzed the role of ROS in BPA promoting the migration and invasion of DLD1 cells. As shown in **Figures 5A, B**, compared to the control group, the cell migration ability of the NAC + BPA group was 125.96%, which was lower than that of the BPA-alone treatment group (225.86%). A similar trend of change was also observed in the invasion results, where the NAC pretreatment decreased BPA-induced elevation in invasion by 45.52% (**Figures 5C, D**). These results showed that BPA can induce the migration and invasion of DLD1 cells and is highly correlated with ROS production. Next, the effects of NAC on the expression of critical mediators of the HIF-1α/VEGF/PI3K/AKT axis were analyzed. As shown in **Figure 5E**, NAC pretreatment significantly reduced the mRNA expression levels of HIF-1α, VEGF, VEGFR1, VEGFR2, PI3K, and AKT in BPA-exposed DLD1 cells. Furthermore, because of the NAC pretreatment, the protein expressions of the above genes were significantly inhibited (**Figures 5F, G**). These results indicated that the promoting effects of BPA on the migration and invasion of CC cells can be achieved by enhancing ROS production and then activating the HIF-1α/VEGF/PI3K/AKT axis.

### HIF-1α Suppression Inhibits BPA-Induced Migration and Invasion of DLD1 Cells

**Figure 6:**
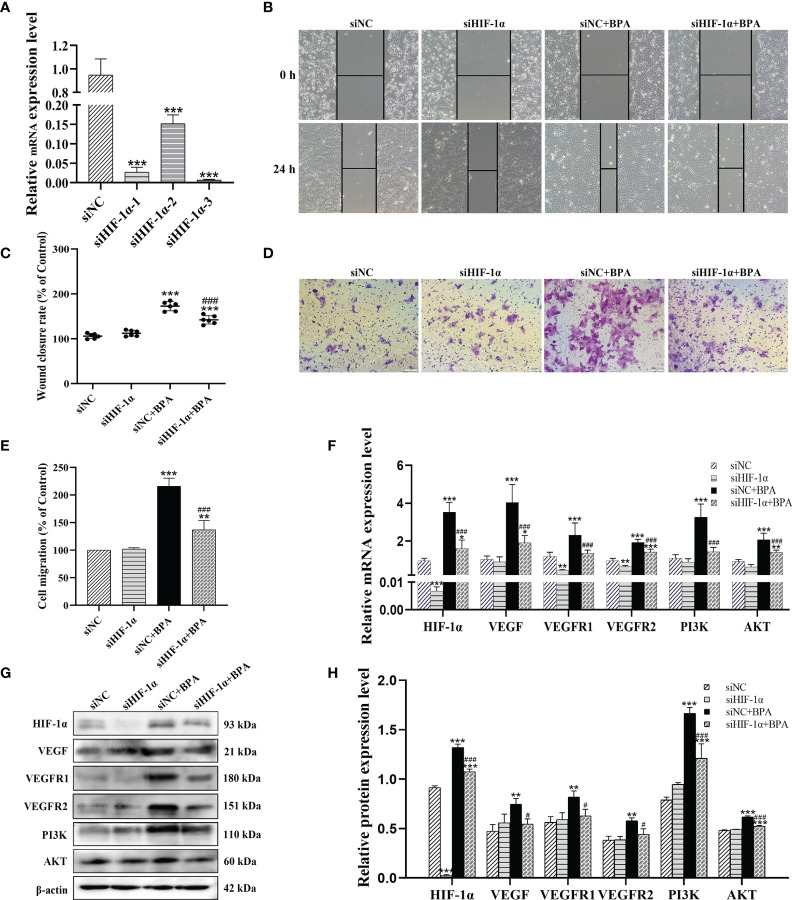
HIF-1α knockdown attenuated the BPA-induced increases in glycolysis, migration, invasion, HIF-1α/VEGF and PI3K/AKT signaling pathway related biomarkers in DLD1 cells. **(A)** Results of qRT-PCR analysis of siHIF-1α inhibition efficiency in DLD1 cells after transfection for 24 h, n = 6. Representative pictures of cell migration **(B)** and invasion **(D)**. Quantitative analysis of cell migration **(C)** and invasion **(E)** rate, n = 6. The mRNA **(F)** and protein **(G)** expression of HIF-1α/VEGF and PI3K/AKT signaling pathway-related biomarkers in DLD1 cells. **(H)** Quantitative analysis of protein expression associated with HIF-1α/VEGF and PI3K/AKT signaling pathway by ImageJ Software, n = 3. Values are expressed as means ± SD, compared with the control group, *p < 0.05, **p < 0.01, ***p< 0.001. Compared with the 1.00E-6 BPA group, ^#^ *p*< 0.05, ^###^ *p* < 0.001.

To verify the role of HIF-1α in the effects of BPA on the migration and invasion of CC cells, we synthesized three knockdown sequences of HIF-1α to intervene the DLD1 cells. The HIF-1α mRNA levels in siHIF-1α-treated cells, especially siHIF-1α-3, were significantly lower than those in the siNC group after transfection for 24 h (**Figure 6A**); thus, we chose siHIF-1α-3 for transfection in the following experiment. Then, we transfected siHIF-1α and siNC into DLD1 cells to detect the migration and invasion of DLD1 cells with or without 10^-4^ mM BPA exposure. The results showed that siHIF-1α significantly inhibited the increase in cell migration (**Figures 6B, C**) and invasion (**Figures 6D, E**) caused by BPA exposure. In addition, we further assessed the transcriptional and translational levels of the genes involved in VEGF/PI3K/AKT signaling pathways. When the cells were transfected with siHIF-1α, the mRNA (**Figure 6F**) and protein (**Figures 6G, H**) content of HIF-1α, VEGF, VEGFR1, VEGFR2, PI3K, and AKT was decreased significantly compared to the BPA-alone treatment group, which indicated that VEGF/PI3K/AKT signaling pathways can be inhibited after suppressing HIF-1α in BPA-exposed DLD1 cells. The above results demonstrated that BPA promoted cell migration and invasion by activating VEGF/PI3K/AKT signaling pathways in a HIF-1α-dependent manner.

## Discussion

The mass production and wide application of BPA seriously affect human beings’ health and threaten the abundance of environmental species. BPA can increase the incidence and susceptibility of various cancers, including breast, ovary, prostate, and even colon cancer (7, 19). Low doses of BPA cause resistance to classical chemotherapeutics in multiple types of cancer cells (20). Our results showed that the BPA content in the serum of CC individuals was significantly higher than that in healthy donors. In addition, BPA exposure caused increased expression of NOX-and ETC-related genes in CC cells, and the excess ROS generation was accompanied by oxidative stress occurrence. In contrast, NAC and siHIF-1α reduced the invasion and migration of CC cells by down-regulating ROS overproduction and inhibiting the activation of the HIF-1α/VEGF/PI3K/AKT axis, respectively.

Considerable literature has reported oxidative stress and adverse reactions induced by various doses of BPA. Although the BPA dose used in this study was not cytotoxic, a significant generation of ROS production was still observed (21). The results of the present study indicated that even low BPA nanomolar concentrations can perturb the balance between ROS and anti-oxidative defense processes in CC cells. Given the evidence that intracellular ROS is mainly produced by ETC and NOX (22), we further detected the gene expression levels of ETC-and NOX-related indicators in DLD1 cells exposed to BPA, and the results confirmed that BPA-induced ROS production was jointly driven by ETC and NOX. Furthermore, Beg et al. used molecular docking to confirm that BPA and its nine analogs bind to thyroxine-binding globulin and thyroid hormone receptors to exert toxicity of endocrine disruption (23). Our AutoDock results reported high-affinity docking poses for each ligand-binding-site pair. PyMol visualized the docking view of BPA in the binding site of NOX and ETC, wherein the binding pose with NOX subunits was closest to the result obtained from Sun et al. (24), hence validating our molecular docking approach. The docking results also provided additional evidence for the BPA-regulated ETC complex, as well as the explanation about the structural characteristics of its binding and activity with ETC protein. ROS is not only a cytotoxic factor but also a signal regulator of tumor progression. ROS-induced oxidative stress is related to tumor migration and invasion. According to the literature, an increase in the ROS level accelerates the migration of CRC cells and the formation of vasculogenic mimicry (25). The migration and invasion of human pancreatic cancer cells are significantly inhibited after reducing ROS production (26). High levels of NOX4/ROS are associated with enhanced motility/migration in lung cancer cells (27). Suppressing the activity of ETC complex effectively inhibits the migration and invasion of ovarian cancer cells (28). Previous evidence has shown that BPA-induced ROS promotes the migration of lung cancer A549 (29). Our observations indicated that low-dose BPA can promote malignant migration and invasion of CC cells. The intervention of the ROS scavenger NAC further confirmed that the BPA-induced migration and invasion of DLD1 cells were positively correlated with ROS production.

The excessive generation of ROS can strengthen the stability of HIF-1α and then promote its binding with hypoxia-responsive elements to induce target gene transcription (30). In our study, a double-positive feedback loop comprised ROS and HIF-1α, as excessive ROS was generated in BPA-treated CC cells, and HIF-1α had a significantly high expression in treated cells compared to non-treated cells, and NAC pretreatment significantly reversed this phenomenon. The results suggest that BPA can promote CC progression by activating HIF-1α expression through accumulated ROS. In the context of ROS overproduction, stably and continuously expressed HIF-1α activated various signal pathways, which promoted the occurrence and progress of tumors, such as migration and adhesion (31). Many studies have confirmed that VEGF is the target gene of HIF-1α (32). Classical research has mainly concentrated on VEGF binding with VEGFR on the endothelial cells by paracrine in tumor. They then activate various classical signal pathways to facilitate cancer progression by angiogenesis (33). With the detailed research on VEGF, VEGF was found to bind with its VEGFR on the cell surface to stimulate cell migration enhanced by signal transport, irrespective of the cell types (tumor and endothelial cells) (34). Nevertheless, the mechanism of VEGF function is complex in tumors. On one hand, a tumor cell-targeted downstream signals lead to an increase in tumor invasion and migration through the autocrine VEGF pathway (35). On the other hand, part of the tumor can recruit the protein tyrosine phosphatase 1B by strengthening MET/VEGFR2 heterozygote to inhibit tumor migration (36). The VEGF function requires further explanation in colon cancer. Fan et al. verified that VEGFR exhibits weak expression in CC samples, compared to the significantly increased expression in patients with liver metastasis. Note that VEGF combined with VEGFR1 promotes tumor metastasis through the autocrine pathway (37). The other study reported that VEGFR1 and VEGFR2 are both expressed in CC samples, and autocrine VEGF binds with both receptors to stimulate the malignant alteration of tumor cells (38). Our study found that BPA increases the transcription and translation levels of HIF-1α, VEGF, VEGFR1, and VEGFR2 in DLD1. PI3K/AKT was confirmed as a canonical signaling pathway downstream of VEGF. PI3K/AKT activation was found to promote cell migration and invasion in melanoma (39) and endometrial cancer cells (40). In addition, advanced glycation end products induce proliferation, invasion, and EMT of human SW480 CC cells through the PI3K/AKT signaling pathway (41). Our results showed that HIF-1α knockdown inhibited the migration and invasion and decreased the expression levels of VEGF, VEGFR1, VEGFR2, PI3K, and AKT in BPA-exposed CC cells, indicating that a low dose of BPA can activate HIF-1α to stimulate a high expression of VEGF, thereby targeting VEGFR to induce the PI3K/AKT signaling pathway function to accelerate tumor metastasis.

EMT, as an attractive factor for metastatic tumor, was reported to be directly involved in the migration risk of CRC cells. However, a surprising result in our experiment was that BPA has no effect on EMT biomarkers’ expression level, indicating that EMT is not essential for the migration of CC cells under the dosage of BPA exposure. Herein, the main reasons for the discrepancy are the exposure dose of BPA, the concentration of FBS, and the difference in the cell lines. In this study, the exposure concentrations of BPA were 1 × 10^-4^ and 1 × 10^-5^ mM, while in previous study, 1 × 10^-2^ mM BPA significantly increased the EMT of A549 cells (42). When the BPA concentration is below 1 × 10^-2^ mM, biomarkers of epithelial–mesenchymal transition might become insensitive. The content of FBS in this study was 2%, compared to the value of 5% in previous studies (42). Furthermore, SW480 CRC cells treated with BPA modulated the protein profiles and promoted the metastasis of CRC cells through EMT induction (6), whereas no effects were observed on EMT in DLD1 cells under BPA exposure. Note that the enhanced tumor cell migration might not be completely related to EMT and was probably caused by actin and sometimes by movement protein change (34). According to the literature, the production amount of ROS was accumulated rapidly under the condition of BPA stimulation, but the increase in ROS might activate the downstream signal pathway to inhibit the occurrence of EMT (43). The exact mechanism remains unclear and requires further research.

## Conclusion

**Figure 7:**
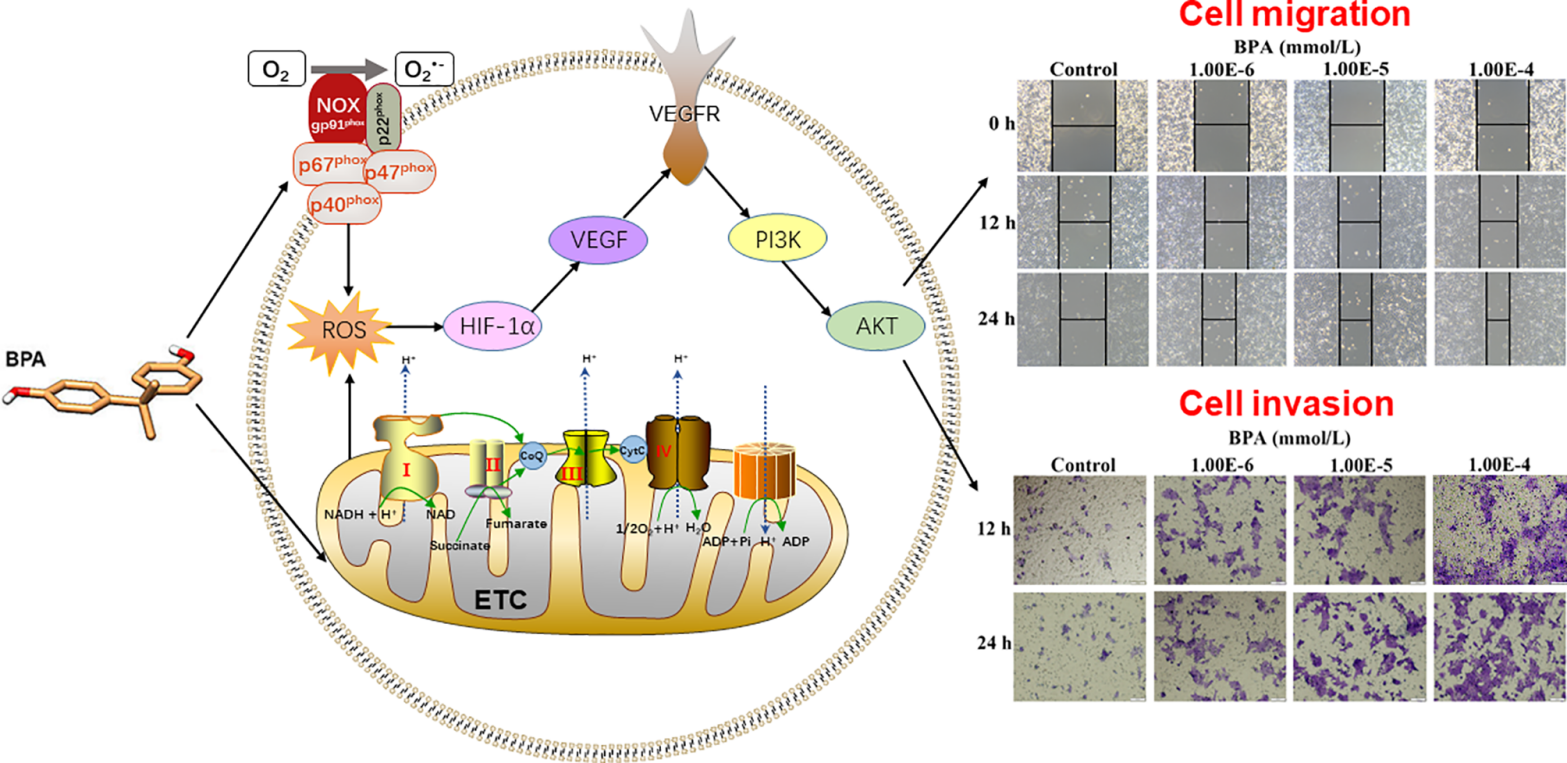
Graphical abstract.

The present study demonstrated that the BPA concentration in the serum of CC individuals is significantly higher than that in healthy donors. Mechanistically, low-nanomolar BPA dual-targeting NOX and ETC induces ROS production, which activates the HIF-1α/VEGF/PI3K/AKT axis to promote the migration and invasion of human CC cells (**Figure 7**). These findings might be helpful in clarifying the underlying CC mechanisms caused by BPA exposure and fully assessing its health risks. However, in-depth mechanisms, biomarkers, and *in vivo* detection need to be further studied.

## Data Availability Statement

The raw data supporting the conclusions of this article will be made available by the authors, without undue reservation.

## Ethics Statement

The studies involving human participants were reviewed and approved by The Ethical Committees of Harbin Medical University Cancer Hospital. The patients/participants provided their written informed consent to participate in this study.

## Author Contributions

TX: Methodology, Data curation, Visualization, Formal analysis, Writing–original draft. JG: Methodology, Formal analysis, Investigation, Data collation. BZ: Methodology, Investigation, Data curation. CS: Methodology, Investigation, Data curation. QZ: Formal analysis, Data curation. YL: Methodology, Resources, Supervision, Writing–review & editing. BC: Supervision, Conceptualization, Writing–review & editing, Funding acquisition. All authors contributed to the article and approved the submitted version.

## Funding

This work is supported by Nn10 Program of Harbin Medical University Cancer Hospital (Nn102017-02), Heilongjiang Sunshine Health Foundation (H21L0802), Haiyan Foundation of Harbin Medical University Cancer Hospital (JJZD2022-17), Beijing Yanchuang Foundation (ZLKY-2021112902).

## Conflict of Interest

The authors declare that the research was conducted in the absence of any commercial or financial relationships that could be construed as a potential conflict of interest.

## Publisher’s Note

All claims expressed in this article are solely those of the authors and do not necessarily represent those of their affiliated organizations, or those of the publisher, the editors and the reviewers. Any product that may be evaluated in this article, or claim that may be made by its manufacturer, is not guaranteed or endorsed by the publisher.
