# Supplementary material for: Bisphenol A Promotes the Progression of Colon Cancer Through Dual-Targeting of NADPH Oxidase and Mitochondrial Electron-Transport Chain to Produce ROS and Activating HIF-1α/VEGF/PI3K/AKT Axis
Source: Front Endocrinol (Lausanne). 2022 Jul 4;13:933051. doi: 10.3389/fendo.2022.933051 (PMC9289207; doi:10.3389/fendo.2022.933051)
Supplement: Supplementary file 1 [file DataSheet_1.docx]

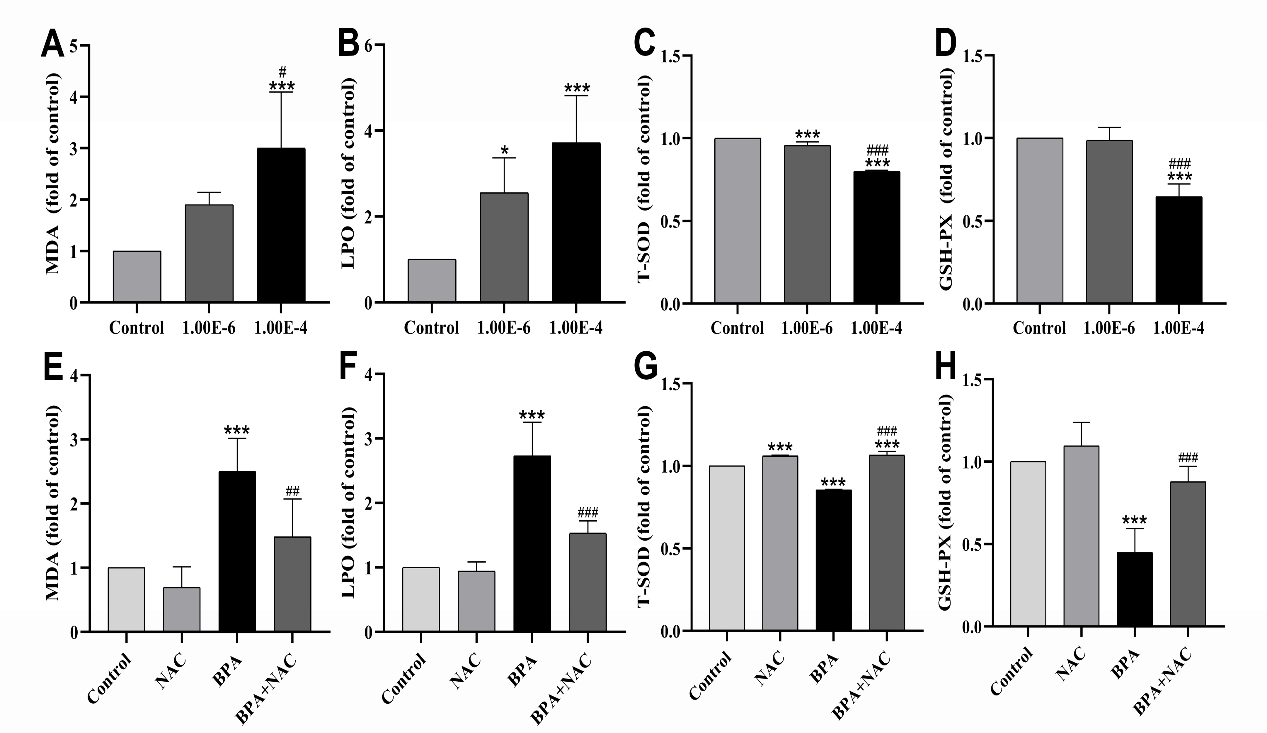


S-Fig.1 MDA (A), LPO (B), T-SOD (C) and GSH-PX (D) were evaluated in DLD1 cells, n = 6. DLD1 cells were treated with 1 × 10^-4^ mM BPA for 24 h in the presence or absence of the ROS inhibitor NAC. The level of MDA (E), LPO (F), T-SOD (G) and GSH-PX (H), n = 6. Values are expressed as means ± SD, compared with the control group, **p* < 0.05, ***p* < 0.01, ****p* < 0.001. Compared with the 1.00E-6 BPA group, ^#^*p* < 0.05, ^###^*p* < 0.001, ns: no significance.


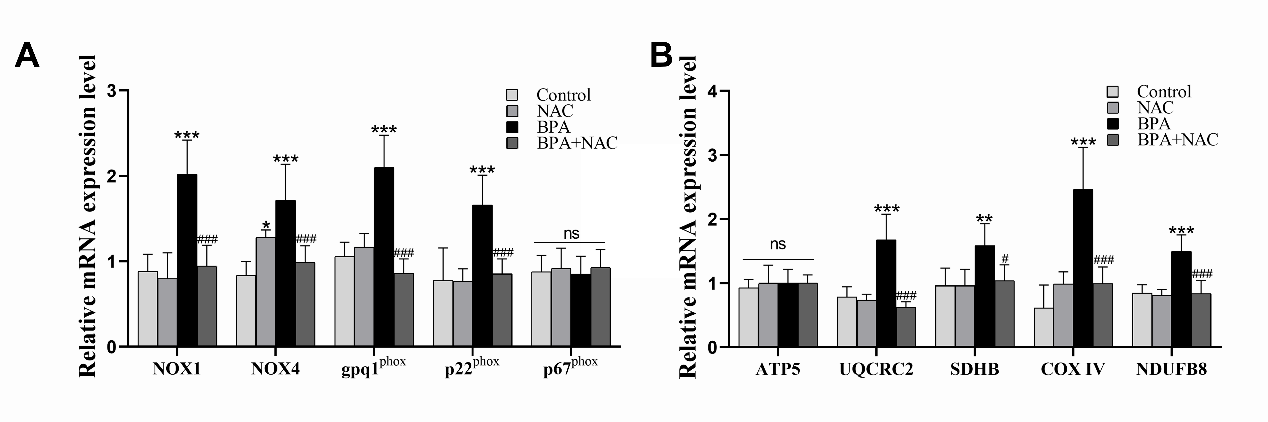
S-Fig.2 DLD1 cells were treated with 1 × 10^-4^ mM BPA for 24 h in the presence or absence of the ROS inhibitor NAC. The mRNA expression levels of mitochondrial respiratory chain complex (A) and NOX subunits (B), n = 6. Values are expressed as means ± SD, compared with the control group, **p* < 0.05, ***p* < 0.01, ****p* < 0.001. Compared with the 1.00E-6 BPA group, ^#^*p* < 0.05, ^###^*p* < 0.001, ns: no significance.

**Supplement-Table 1.** Primer pairs used in qRT-PCR.

| Gene | Primer | Sequence (5’→3’) |
| --- | --- | --- |
| NOX1 | Forward | GAAGTGGCTGTGCTGGTTGGAG |
|  | Reverse | GTCACGATGTCAGTGGCCTTGTC |
| NOX4 | Forward | CCAAGCAGGAGAACCAGGAGATTG |
|  | Reverse | CAGGCCAGGAACAGTTGTGAAGAG |
| gp91^phox^ | Forward | ACCGGGTTTATGATTCCACCT |
|  | Reverse | GATTTCGACAGACTGGCAAGA |
| p22^phox^ | Forward | GTACATGACCGCCGTGGTGAAG |
|  | Reverse | GCTTGATGGTGCCTCCGATCTG |
| p67^phox^ | Forward | AGCTCTCCGCAGTCCGACATC |
|  | Reverse | TCACACCACAGAGTCAGGCAGTAG |
| ATP5 | Forward | CTCCGGAATCGGCCCTGTC |
|  | Reverse | GTCCTCCAGATGTCTGTCGCT |
| UQCRC2 | Forward | CCTGCGGGGTGATGTTGATA |
|  | Reverse | CAGCTACTTCCCAACGACGA |
| SDHB | Forward | CACTCTAGCTTGCACCCGAA |
|  | Reverse | CGTCCAGTTTCTCACGCTCT |
| COX IV | Forward | TGGCGGCAGGTGTACATTTT |
|  | Reverse | AGTCTTCGCTCTTCACAACACT |
| NDUFB8 | Forward | TGGATACATCCCCCACACCT |
|  | Reverse | AGAAGTGGTGCCTACACACAG |
| HIF-1α | Forward | TGAGGGACGGAGATTTTCTTCA |
|  | Reverse | CAATATCCAAATCACCAGCATCC |
| VEGF | Forward | ACATCACCATGCAGATTATGCG |
|  | Reverse | CTCCAGGGCATTAGACAGCA |
| VEFGR1 | Forward | AAGCTTTTATATCACAGATGTGCC |
|  | Reverse | GCCTTTTTGTTGCAGTGCTCA |
| VEGFR2 | Forward | TCCTTCTCTAGACAGGCGCT |
|  | Reverse | AGGCTCCAGTGTCATTTCCG |
| PI3K | Forward | AAGAGCCCCGAGCGTTTCTG |
|  | Reverse | GCCTCACGGAGGCATTCTAA |
| AKT | Forward | TGTCTCGTGAGCGCGTGTTTT |
|  | Reverse | CCGTTATCTTGATGTGCCCGTC |
| β-actin | Forward | CGCACCACTGGCATTGTCAT |
|  | Reverse | TTCTCCTTGATGTCACGCAC |

**Supplement-Table 2.** **Primary** **antibodies reference** **catalog**

| **Antibodies** | **Cat. No** | **Manufacturer** | **Diluted multiples** |
| --- | --- | --- | --- |
| Twist | WL00997 | Wanlei, Shenyang, China | 1:500 |
| E-cadherin | WL01482 | Wanlei, Shenyang, China | 1:750 |
| Smad2 | WL02286 | Wanlei, Shenyang, China | 1:750 |
| HIF-1α | WL01607 | Wanlei, Shenyang, China | 1:500 |
| VEGF | WL00009b | Wanlei, Shenyang, China | 1:750 |
| VEGFR1 | WL03690 | Wanlei, Shenyang, China | 1:500 |
| VEGFR2 | WL02294 | Wanlei, Shenyang, China | 1:500 |
| PI3K | WL03380 | Wanlei, Shenyang, China | 1:1250 |
| AKT | WL0003b | Wanlei, Shenyang, China | 1:750 |
| β-actin | AC026 | ABclonal, Wuhan, China | 1:20000 |
